# Emotional Dysfunction in Parkinson’s Disease

**DOI:** 10.3233/BEN-2011-0329

**Published:** 2011-08-29

**Authors:** Lee X. Blonder, John T. Slevin

**Affiliations:** ^1^Sanders-Brown Center on Aging and Departments of Behavioral Science and NeurologyUniversity of Kentucky College of MedicineLexingtonKYUSA; ^2^Department of NeurologyUniversity of Kentucky College of Medicineand Neurology Service Veterans Affairs Medical CenterLexingtonKYUSA

**Keywords:** Parkinson's disease, emotion, depression, anxiety, neuropsychological impairment

## Abstract

In addition to motor symptomatology, idiopathic Parkinson’s disease is characterized by emotional dysfunction. Depression affects some 30 to 40 percent of Parkinson patients and other psychiatric co-morbidities include anxiety and apathy. Neuropsychological and neuroimaging studies of emotional dysfunction in Parkinson patients suggest abnormalities involving mesolimbic and mesocortical dopaminergic pathways. There is also evidence suggesting that the interaction between serotonin and dopamine systems is important in the understanding and treatment of mood disorders in Parkinson’s disease. In this review we discuss the neuropsychiatric abnormalities that accompany Parkinson's disease and describe their neuropsychological, neuropharmacologic, and neuroimaging concomitants.

## 1. Introduction

Idiopathic Parkinson's disease (PD) is arguably the second most frequently occurring neurodegenerative disorder in the United States, affecting approximately 1 million individuals. The prevalence of idiopathic PD is approximately 9.5/1000 for persons $\geqslant$ 65 or 70 years of age [55]. The three cardinal features of this disease are bradykinesia (slowness and poverty of movement), muscular rigidity, and resting tremor. As the illness progresses, postural instability invariably occurs. These motor symptoms are often accompanied by impairments in emotional and cognitive processing. Some evidence suggests that motor and non-motor symptoms in idiopathic PD are related to a larger pathologic process involving a progressive caudal to rostral aggregation of alpha-synuclein in select nerve cells [17]. However, further research is required to confirm this [21].

One of the pathological hallmarks of idiopathic PD is the selective loss of dopaminergic neurons in the pars compacta region of the substantia nigra. This loss results in dysregulation of neuronal activity in the basal ganglia and its thalamo-cortico-brainstem connections [104]. Idiopathic PD is also associated with loss of dopaminergic neurons in the ventral tegmental area of the midbrain [117]. The major dopaminergic pathways implicated in idiopathic PD are (1) the nigrostriatal pathway that extends from the pars compacta of the substantia nigra to the striatum (caudate and putamen) and (2) the mesolimbic and mesocortical pathways that originate in the ventral tegmental area. The mesolimbic pathway projects to the nucleus accumbens, amygdala, olfactory nuclei, entorhinal cortex, and hippocampus. The mesocortical pathway extends to prefrontal and cingulate cortices [103]. The contribution of the ventral tegmental area to the mesocorticolimbic dopaminergic pathways was established in rodent models. Subsequent studies in non-human primates have suggested that the midbrain origin of these pathways may be more widespread [165].

In the last few years, neuroimaging research in humans has corroborated animal tracing studies and shed further light on basal ganglia circuitry and its relevance to emotional dysfunction in PD. In a meta-analysis of 126 functional neuroimaging studies, Postuma and

ISSN 0953-4180/11/$27.50 © 2011 – IOS Press and the authors. All rights reserved

Dagher [116] found evidence to support the tripartite model of basal ganglia connectivity proposed by Alexander et al. [3]. This model divides the basal ganglia into motor, associative, and limbic areas. Most pertinent to this review, Postuma and Dagher [116] found co-activation of the ventral striatum (the nucleus accumbens and ventral caudate), and the medial temporal lobe that includes the hippocampus and amygdala. More recently, Di Martino et al. [36] conducted a resting state functional magnetic resonance imaging (fMRI) study of basal ganglia connectivity in humans. Their results were largely consistent with the meta-analysis of Postuma and Dagher [116], and, in addition, they found evidence of functional connectivity between (1) the ventromedial caudate and the lateral orbitofrontal cortex (OFC) and (2) the nucleus accumbens and the medial OFC/anterior cingulate. The OFC and the anterior cingulate are implicated in emotion regulation, reward processing, and decision making in humans [61].

Given the functional connectivity of the basal ganglia, it is not surprising that PD patients often have emotional disorders. It is increasingly recognized that affective syndromes may be as disabling as the classic Parkinsonian motor symptoms, if not more so. In the following review, we describe the neuropsychiatric syndromes associated with idiopathic PD and discuss their neuropharmacologic and neuropsychological concomitants within the context of pathologically altered midbrain and pontine rostral projections.

## 2. Depression in Parkinson's disease

Depression is a serious and relatively common complication of Parkinson's disease, although estimates of prevalence vary due to methodological factors [24,27,31,54,77,85,86,98,101,106,120,124,136,143,147,155,157]. In a report published in 1992, Cummings [31] concluded that approximately 40 percent of Parkinson patients suffer from depression and about half of those suffer from major depressive disorder (MDD). Slaughter et al. [136] reviewed 45 studies published between 1922 and 1998 and found that on average, about 31 percent of Parkinson patients are depressed. Veazey et al. [155] reported that rates of depression in published studies of PD range from 7 percent to 76 percent. A study of male veterans with PD found that 19 percent have a diagnosis of depression; with MDD accounting for 21.3 percent and minor depression for the other 78.7 percent of cases [24]. More recently, Reijnders et al. [120] conducted a meta-analysis of depression prevalence in PD based on 36 selected studies and calculated that 17 percent of Parkinson patients suffered from MDD, 22 percent had minor depression, and 13 percent were dysthymic. In those studies using criteria set forth in the Diagnostic and Statistical Manual (DSM) of the American Psychiatric Association, the prevalence rates were somewhat lower [120].

The major contributors to depression in PD (dPD) are thought to be depletion of brain catecholamines and serotonin and dysregulation of fronto-subcortical connections that regulate mood. Several studies have shown that depression may precede the diagnosis of PD, suggesting that depression is either an early symptom or perhaps even a risk factor [68,100,101,132]. Arabia et al. [4] reported an elevated incidence of depression and anxiety in relatives of Parkinson patients, further indicating that genetically-mediated neuropathological commonalities account for psychiatric co-morbidities. Studies of allelic variation in neurotransmitter transporter genes thought to play a role in Parkinsonian depression have been inconsistent. Both Menza et al. [92] and Mossner et al. [95] found an association between dPD and a short allele variant of the serotonin transporter gene. However, a recent analysis of dopamine and serotonin transporter genes in PD patients with a history of depression showed no significant genotype or haplotype associations with depression [41]. The possibility that Parkinsonian depression may have a reactive component should not be discounted, and some evidence supports this [54,76]. However, the association between depression and severity of illness is weak, and depressed PD patients have little guilt or shame [75].

### 2.1. Assessment of depression in Parkinson's disease

Variations in estimations of dPD prevalence are due in part to heterogeneity in diagnostic criteria and methods of assessment. There is widespread agreement in the psychiatric community that the cardinal symptoms of MDD are affective in nature: i.e., persistent depressed mood and markedly diminished interest or pleasure in activities (anhedonia). However, according to the criteria set forth in the DSM IV, other symptoms factor into the diagnosis, including somatic and vegetative complaints such as weight loss, sleep disturbance, and/or fatigue. Screening instruments vary in the proportion of ideational, somatic, and vegetative symptoms they include. For example, the Geriatric Depression Scale (GDS) largely excludes references to somatic indicators while the Beck, Hamilton, and Zung depression scales include them (see Schrag et al. [131] for detailed comparisons). Furthermore, the DSM IV stipulates that those symptoms that are clearly caused by a general medical condition be excluded in making the diagnosis. The inclusion or exclusion of somatic and vegetative indicators have proven problematic in diagnosing dPD given that PD patients often have motor slowing, sleep disturbances, loss of energy, weight changes, and change in appetite (all criteria for MDD) whether they are depressed or not. Elimination of these indicators in the assessment of dPD may result in a reversal of diagnosis from depressed to non-depressed [15].

Lack of clear guidelines pertaining to the diagnosis of depression in PD prompted the National Institute of Neurologic Diseases and Stroke (NINDS) and the National Institute of Mental Health (NIMH) to convene a Work Group to review the data and make recommendations [81]. The work group determined that the strict DSM criteria for diagnosing depression and dysthymia are difficult to use in Parkinson patients and may result in under-diagnosis of the disorder. They recommended eliminating the DSM exclusion criterion "due to the effects of a general medical condition." This approach is designed to increase the likelihood of identifying cases of clinically significant depression and is the consensus panel's recommended standard to be used in clinical research on depression and Parkinsonism. Thus it is advised that all symptoms be considered as related to depression, regardless of their overlap with PD or other medical conditions. Starkstein et al. [141] validated these expanded criteria in a clinic-based study of 173 PD patients. They found that 30 percent of the sample met DSM IV criteria for MDD and 20 percent for dysthymia.

Within the last few years a number of methodologically rigorous studies have examined the validity of depression rating scales in Parkinson patients. For example, several investigators have found that both the 30 and 15-item versions of the GDS are valid in screening for depression in PD [44,87,131,153,162]. Weintraub et al. [162] showed that the 15-item version is valid in Parkinson patients both under and over the age of 65. The GDS is easy to administer in the clinic, contains few somatic indicators of depression, and uses a simple yes/no response format, making it highly suitable for Parkinson patients. However, a disadvantage is its somewhat low sensitivity to depression severity [131].

Other instruments that have proven valid in screening for Parkinsonian depression include the Beck Depression Inventory, Hamilton Depression Rating Scale, Montgomery-Asberg Depression Rating Scale, Zung Depression Inventory, and the Cornell Scale for Depression in Dementia [39,69,131,135,153,156,164]. The choice of a scale to screen and diagnose dPD must be based in part on time constraints and the availability of skilled examiners. Scales that are clinician-administered such as the Hamilton Depression Rating Scale and the Montgomery-Asberg Depression Rating Scale require specialized training to insure validity and reliability. Self-report scales such as the GDS and the Beck Depression Inventory can be used in a broader range of settings as they do not require administration by trained mental health professionals and are easy to score. Given the prevalence and seriousness of Parkinsonian depression, routine use of one of these validated depression screening measures should be part of every PD patient's clinical assessment. This is especially important in view of survey results showing that many PD patients believe that depression is a normal response to the disease process and would be reluctant to initiate a discussion of it with their healthcare provider [123].

### 2.2. *Neuroimaging of depression in Parkinson's disease*

#### 2.2.1. *Positron emission tomography*

During the last thirty years, several positron emission tomography (PET) studies have examined the neural substrates of Parkinsonian depression. Most of these studies show hypometabolism and catecholamine abnormalities in pre-frontal regions, the anterior cingulate cortex, and portions of the basal ganglia and limbic system. In one of the first such studies, Mayberg et al. [84] found that metabolism in the caudate and inferior OFC was lower in depressed compared with both non-depressed Parkinson patients and control subjects. The magnitude of the metabolic change in the inferior OFC was inversely related to the severity of depression. Ring et al. [124] found bilateral decreases in blood flow in the anterior medial prefrontal cortex [Brodmann's Area (BA) 9] and the anterior cingulate (BA 32) of both depressed PD and psychiatric patients compared with non-depressed PD and control participants. Depressed PD patients demonstrated hypoperfusion relative to depressed psychiatric patients in BA 10 of the right medial frontal cortex. Although the significance of this finding is unclear, BA 10 is important in the processing of positive emotion and in attending to one's own emotions and the mental states of others [53,59].

PET studies of neurotransmitter activity in Parkinsonian depression suggest loss of dopamine and norepinephrine innervation in the limbic system. Remy et al. [122] used [11C]RT1-32 PET to study dopamine and norephinephrine transporter binding in depressed and non-depressed Parkinson patients. They found lower binding in dPD patients in the locus ceruleus, caudate, anterior cingulate, thalamus, amygdala, and ventral striatum. Boileau et al. [16] performed PET studies of dopamine D3 receptor binding in mild to moderate, drug-naïve PD patients and showed reduced D3 receptor activity that correlated with negative affect on mood scales [16]. D3 receptors tend to be located in the limbic system and globus pallidus and are implicated in affect regulation and reward seeking behavior (see below).

### 2.3. Transcranial sonography

Transcranial sonography (TCS) has been used to visualize deep brain pathology in Parkinsonian depression [13,159]. Initially, Becker et al. [10] found that TCS was capable of identifying substantia nigra hyperechogenicity as a biomarker of Parkinson's disease. Hyperechogenicity is thought to reflect increases in iron concentrations and is both reliable and sensitive in differentiating idiopathic PD patients from neurologically normal individuals and those with atypical Parkinsonian syndromes [12,89]. Using TCS and MRI, Berg et al. [13] found reduced mesencephalic midline echogenicity in depressed versus non-depressed PD patients, affirming limbic system abnormalities in dPD. More recently, Walter et al. [159] compared healthy controls, PD patients with and without depression, and depressed psychiatric inpatients without PD. They found marked hyperechogenicity of the substantia nigra in 87 percent of depressed PD patients, 69 percent of non-depressed PD patients, 40 percent of depressed psychiatric patients, and 13 percent of healthy controls. They also found significant reductions in echogenicity in the brainstem raphe among depressed patients irrespective of a diagnosis of PD. The brainstem raphe is rich in serotonin-producing neurons, some of which project to the substantia nigra and modulate dopamine transmission [1,37]. Finally, the co-occurrence of pronounced hyperechogenicity of the substantia nigra and reduced echogenicity in the brainstem raphe was associated with a history of depression preceding a diagnosis of PD. Taken together, these results indicate that abnormalities in the nigrostriatal dopaminergic system and the serotonergic brainstem raphe play a role in both Parkinsonian and idiopathic depression. Furthermore, pathology in the nigrostriatal dopaminergic system may contribute to sub-clinical motor dysfunction in patients with idiopathic depression.

The findings of Walter et al. [159] raise important issues regarding the neurobiological underpinnings of Parkinsonian depression. In particular, this study is the first to associate the presence of depression preceding PD with TCS abnormalities, although past studies have found that depression is associated with an elevated risk of developing PD [68,100,101,132]. If future research confirms an association between hyperechogenicity of the substantia nigra and reduced echogenicity in the brainstem raphe in depressed patients who subsequently develop PD, then this pattern of TCS abnormalities could serve as a biomarker in preventative intervention trials. To identify further the mechanisms underlying Parkinsonian depression, as well as the optimal target population to screen, future research might also examine the association between TCS abnormalities and serotonin transporter polymorphisms in depressed PD patients.

### 2.4. Magnetic resonance imaging

Several studies have used structural and functional MRI to study Parkinsonian depression. Matsui et al. [82] compared depressed versus non-depressed PD patients using diffusion tensor imaging (DTI). They found reduction in fractional anisotropy in the anterior cingulate of depressed PD patients. Fractional anisotropy measures the directional dependence of water diffusion and is considered a sensitive indicator of the presence and degree of histological abnormality in white matter tracts [82].

Feldmann and collaborators [46] conducted a voxel-based morphometry (VBM) study in depressed versus non-depressed PD patients and showed reductions in gray matter density in the left OFC, bilateral rectal gyrus, and right temporal regions of the depressed group. They also found a negative correlation between depression severity and gray matter density in the OFC, right medial temporal gyrus, right parahippocampal gyrus, anterior cingulate, and cerebellum. These results support the PET studies cited above that describe OFC and cingulate abnormalities in depressed PD patients. The cerebellar finding is consistent with psychiatric research that shows cerebellar gray matter reduction and resting state cerebellar fMRI abnormalities in patients with MDD [78,110]. Cerebellar involvement in mood regulation may occur via its connections with the limbic system [130].

Using fMRI, Cardoso et al. [22] found decreased activation in the medial prefrontal cortex and the left mediodorsal thalamus of depressed versus non-depressed PD patients during a facial affect perception task. Subsequent VBM studies targeting the thalamus showed increased volume in the mediodorsal thalamic nuclei bilaterally in depressed PD patients [22]. The mediodorsal thalamus is a dopamine rich region that receives input from the amygdala, olfactory system, basal ganglia and hypothalamus and projects to the prefrontal association cortex. These results corroborate post-mortem and imaging studies in psychiatric patients that reveal significant enlargement of the amygdala and mediodorsal thalamus in MDD [49,169]. MDD is also associated with reductions in the volume of some subcortical brain regions–particularly in the caudate, putamen, and hippocampus [11,18,50,62,99,171]. Certain genetic polymorphisms in serotonin transporter genes have been identified in psychiatric MDD that may predispose these patients to enlargements or reductions in regional gray matter volume [51,169]. As described above, similar allelic variants have been identified in depressed PD patients [92,95], although we know of no published research linking depression, serotonin transporter polymorphisms, and variations in regional gray matter volume in PD.

Kostic et al. [60] also used VBM to examine the neural substrates of dPD. They found that depressed PD patients had significant reduction in white matter density in the right anterior cingulate and inferior OFC compared with non-depressed PD patients. Furthermore, depression severity correlated significantly with white matter loss in the OFC. Gray matter volume did not differ as a function of mood status in PD. Like the DTI findings of Matsui et al. [82], these white matter abnormalities suggest that Parkinsonian depression is characterized by disconnection of fronto-subcortical circuits. Kostic and collaborator's work [60] contrasts with studies by Feldmann et al. [46] and Cardoso et al. [22] that show specific gray matter changes in association with Parkinsonian depression. The source of these discrepant findings is unclear but one explanation is that they are methodological in nature: due to differences in scales used to diagnose dPD or in MRI/VBM analytic techniques.

In sum, PET, TCS, and MRI studies have found abnormalities in the basal ganglia, limbic system, and frontal lobes associated with dPD. The specific regions most often implicated are the caudate, thalamus, ventral striatum, orbitofrontal cortex, anterior cingulate, and medial prefrontal cortex. The identified abnormalities include hypoperfusion, dysregulation of monoamine neurotransmitters, altered echogenicity on TCS, and gray and white matter changes. VBM and DTI studies indicate that fronto-subcortical connectivity is pathologically altered in Parkinsonian depression. Some evidence suggests overlap between Parkinsonian and psychiatric depression in the pattern of regional brain abnormalities; however few studies have directly compared PD and psychiatric MDD patients.

### *2.5. Neuropsychological studies of depression in Parkinson's disease*

Neuropsychological studies of depression in PD tend to corroborate neuroimaging research in suggesting pre-frontal dysfunction. In particular, dPD patients have significant impairments in memory, verbal fluency, visual confrontation naming, and set-shifting compared with non-depressed PD patients [151,152,154, 163]. Kuzis et al. [63] showed that while depression alone is associated with decrements in verbal fluency and auditory attention, the combination of depression and PD results in further impairments in set shifting and concept formation. Deficits in verbal fluency and working memory appear early in the disease course and are present in mildly depressed PD patients as well as those with moderate to severe depression [30,154], Research suggests that decline in these cognitive functions varies in relationship to the severity of depression [30,143]. For example, Costa et al. [30] showed that Parkinson patients with major depression performed significantly worse than those without depression on measures of episodic and working memory, abstract reasoning, visuospatial abilities, and language. PD patients with minor depression showed some impairment on these tasks, but they did not reach statistical significance. Work by Stevanova et al. [143] indicates that Parkinson patients with dysthymia manifest a similar pattern of deficits to non-depressed PD patients-yet more severe. PD patients with MDD have more extensive impairments in episodic visuospatial memory, spatial working memory, and naming.

Several neuroimaging experiments have investigated brain mechanisms of cognitive dysfunction in PD [74, 83,107], however few have included depression status as a variable in the design. More commonly, brain imaging studies have examined the neural correlates of mood in conjunction with other variables such as cognitive performance and motor function. For example, Broussolle and collaborators [20] utilized PET and event-related potentials to study the contribution of striatal dopamine deficiency to motor skills, memory, executive function, attention, and mood state in non-demented PD patients. They found a close association between striatal dopamine deficiency and locomotor abilities but little relationship with cognitive function or mood, suggesting that abnormalities in these non-motor functions may result from dysfunction in mesocorticolimbic dopamine pathways or non-dopaminergic systems. Rinne et al. [125] found a correlation between [18F] fluorodopa uptake in the caudate and frontal lobes and performance on tests of executive function in patients with PD. Depression severity scores on the Beck Depression Inventory (BDI) did not correlate with [18F] fluorodopa uptake in any region investigated, however. This negative result is difficult to interpret as the number of depressed PD patients is not reported. Mentis et al. [90] conducted multivariate voxel-based analysis of PET data and found that BDI scores correlated with topographic patterns of metabolic activity involving the dorsolateral prefrontal cortex, OFC, medial frontal cortex, and the anterior cingulate. In contrast, visuospatial and mnemonic functions correlated with metabolic activity involving parieto-occipital-temporal and medial temporal regions. The authors suggest that different neural mechanisms underlie cognitive and dysphoric symptoms in PD, however such a conclusion may be premature given that none of the PD patients in this study met criteria for MDD and the test battery did not include standard measures of executive function.

Taken together, these results indicate that neuropsychological dysfunction increases with increasing severity of depression in PD patients and is primarily dysexecutive in nature. Furthermore, it is likely that a common neuropathological process involving the basal ganglia, limbic system, thalamus and their connections with the dorsolateral prefrontal cortex, OFC, and the anterior cingulate – underlies both depression and executive dysfunction in PD. While mesocorticolimbic dopamine abnormalities are probably a principal contributor to this co-morbidity, dysregulation of other neurotransmitter systems may play a role (see below).

### 2.6. *Neuropharmacology of depression in Parkinson's disease*

The neurotransmitter abnormalities that underlie idiopathic PD overlap with those implicated in affective disorders among psychiatric patients. Therefore it is not surprising that PD patients show symptoms of dysphoria. In addition to dopamine, idiopathic PD patients have depletion of brain norephinephrine and serotonin [96,127]. Norepinephrine and serotonin were initially implicated as etiologic agents in depressive mood when hypertensive patients receiving reserpine therapy developed dysphoria [47]. Reserpine depletes central and peripheral catecholamines and serotonin by blocking the vesicular monoamine transporter (VMAT). During the 1950s and 1960s pharmacologists investigating anti-tuberculosis drugs and anti-histamines developed compounds that were later found to elevate mood [112, 129]. The first two such compounds were iproniazid, an inhibitor of the enzyme monoamine oxidase (MAO), and imipramine, a tricyclic antidepressant that inhibits the reuptake of several neurotransmitters but principally norepinephrine. These early findings lead to the catecholamine hypothesis of depression, which posited that reductions in norepinephrine bioavailabity underlie dysphoria [129]. Subsequent studies showed low levels of hydroxyindoleacetic acid (5-HIAA), the acid metabolite of serotonin, in the cerebrospinal fluid of depressed patients and those who committed suicide [28]. In 1971, Wong and his associates at Eli Lilly developed fluoxetine, the first selective serotonin reuptake inhibitor (SSRI) [168]. SSRIs have largely supplanted the earlier MAO inhibitors and tricyclic antidepressants as the first line of treatment for unipolar depression due to their equivalent clinical efficacy and more favorable side-effect profile [79].

As a consequence of these advances in the understanding and treatment of psychiatric depression, research on the neuropharmacology of dPD has focused on abnormalities in monoamine neurotransmitter systems. In particular, Chan-Palay and Asan [23] found extensive loss of norepinephrine neurons in the rostral and caudal portions of the locus ceruleus of dPD patients, while patients who had not been depressed showed relative sparing of caudal neurons. Jellinger and Paulus [57] showed that Parkinson patients with depression had greater cell loss in the dorsal raphe nucleus than did non-depressed Parkinson patients, implicating serotonin abnormalities in the genesis of dPD. A recent postmortem study comparing depressed with non-depressed Parkinson patients indicated a higher prevalence of neuronal loss and gliosis in the locus ceruleus, dorsal motor nucleus of the vagus nerve, and pars compacta of the substantia nigra but not the dorsal raphe nucleus, implicating catecholamine but not serotonin abnormalities in the pathophysiology of Parkinsonian depression [48]. Using single photon emission tomography (SPECT), Murai et al. [96] found significant correlations between dorsal midbrain binding ratios (reflecting the serotonergic raphe system) and neuropsychiatric symptoms in PD patients. Taken together, these findings implicate norepinephrine and serotonin abnormalities in Parkinsonian depression. Discrepancies may be due to variation in methodology, patient characteristics, or to a combination of factors.

There is some evidence that dysphoria associated with PD may be related to abnormal dopamine neurotransmission. Several studies have reported that levodopa medication affects patients' mood. Spigset and von Scheele [138] reported two patients who increased their own dosage of levodopa to 1500–2000 mg/day to induce feelings of euphoria. Maricle et al. [80] found that mood elevation, anxiety reduction, and a corresponding increase in tapping speed were related to levodopa but not to placebo infusion in a sample of Parkinson patients. Torack and Morris [148] found dopamine depletion in the ventral tegmental area of depressed but not of non-depressed Parkinson patients, suggesting that degeneration of dopaminergic terminals in this region may contribute to mood disorders in PD. More recently, Black et al. [14] used PET to study Parkinson patients with mood fluctuations before and after levodopa administration. They found that the response to levodopa in the medial frontal gyrus and posterior cingulate differed between PD patients with mood fluctuations and those with motor fluctuations. Abnormalities in the medial frontal gyrus of dPD patients have been reported in previous studies [14,124]. Black et al. [14] speculate that abnormal dopaminergic modulation of structures that innervate the posterior cingulate (e.g. the caudate, anterior cingulate, or OFC) may contribute to their findings.

### 2.7. Treatment studies

The complexity surrounding the neuropharmacologic substratum of depression in PD is underscored in part by the inconclusive results of treatment studies [155,160,161]. Factors that contribute to this include methodological challenges such as recruitment difficulties and lack of statistical power, failure to employ double-blind randomized placebo-controlled designs, and reliance on open-label or anecdotal evidence [161]. For example, Leentjens et al. [70] studied the efficacy of the SSRI sertraline in the treatment of Parkinsonian depression and found no effect relative to placebo. However, only twelve patients – six per arm – participated in this study. Others have reported that depressed patients taking SSRIs have developed Parkinsonian symptoms or have had aggravation of preexistent symptoms, but such side-effects are uncommon [26,88].

Menza et al. [91] conducted a randomized, placebo-controlled trial in 52 dPD patients comparing the SSRI paroxetine CR, the tricyclic antidepressant nortriptyline, and placebo. They found that nortriptyline was efficacious in the treatment of depression and paroxetine CR was not. Tricyclic antidepressants are known to increase dopamine transmission in the frontal cortex, providing further support for the role of dopamine in Parkinsonian depression [94]. Devos et al. [34] conducted a double-blind, randomized, placebo-controlled study of 48 non-demented Parkinson patients suffering from major depression. They compared desipramine, a tricyclic noradrenergic reuptake inhibitor and citalopram, a SSRI. They found that desipramine resulted in greater improvement on the Montgomery-Asberg Depression Rating Scale (MADRS) compared with both citalopram and placebo after 14 days, but both antidepressants produced significant improvements in MADRS scores after 30 days. The short-term clinical advantage of desipramine was mitigated by a higher frequency of mild adverse events. Some studies in the psychiatric literature suggest that a newer class of anti-depressants, selective serotonin-norepinephrine re- uptake inhibitors (SNRIs), may have slightly greater efficacy than SSRIs without the side-effects noted in tricyclic anti-depressants [108]. At present, there are no published clinical trials of SNRIs in Parkinson depression but a report involving two patients with PD revealed remission from SSRI refractory depression when placed on SNRIs [146]. The "Study of Anti-Depressants in Parkinson's Disease" (SAD-PD), a NINDS-sponsored randomized, double-blind, placebo-controlled clinical trial comparing paroxetine (SSRI) and venlafaxine extended release (SNRI), has been completed but results have not yet been reported (personal communication).

Several studies suggest that dopamine agonists may be of some therapeutic benefit in the treatment of Parkinsonian depression; however few methodologically rigorous clinical trials have been conducted using these drugs. In an early study, high doses of bromocriptine, a predominantly D-2 dopamine receptor agonist, were shown to ameliorate depressive symptoms in a sample of ten Parkinson patients [58]. Pramipexole, a dopamine agonist with affinity for both D2 and D3 receptors, has shown some promise in the treatment of depression, both in psychiatric and Parkinson patients [6,

29,71,72,118,121]. A recent double-blind study found that pramipexole, compared with placebo, improved depressive symptoms in dPD patients with stable motor function [5]. Studies in rodents show that this drug also modifies the spontaneous firing of norepinephrine and serotonin neurons, suggesting that its therapeutic effect may be due in part to modulations of these neurotransmitters [5,25].

### 2.8. *Brain reward system and Parkinsonian depression*

Abnormalities in the "brain reward system" may play a role in Parkinson depression and associated cognitive dysfunction. Numerous studies of reward expectation, principally in the substance abuse literature, suggest that activation of dopaminergic neurons in mesolimbic and mesocortical pathways is critical in producing the pleasurable and reinforcing effects associated with certain stimuli, such as cocaine, nicotine, and monetary reward [43,65]. Furthermore, fMRI studies of reward circuits in healthy adults have identified a link between the dorsolateral prefrontal cortex and the medial frontal cortex during cognitive and motivational components of an n-back working memory task [114]. Functional abnormalities in these regions have been identified in Parkinsonian depression [124]. Several investigators have proposed that depressive disorder is related to dysfunction of dopamine transmission in brain reward pathways [19]. This hypothesis is consistent with the fact that a core symptom of depression is anhedonia, or reduction in the capacity to experience pleasure. In support of this hypothesis, Naranjo et al. [97], Tremblay and Tremblay [149] and Tremblay et al. [150] showed that psychiatric patients with major depression exhibit hypersensitivity to drugs that are known to stimulate brain reward sites. Using fMRI, Tremblay and colleagues [150] found that this hypersensitivity is associated with altered brain activation in the ventrolateral prefrontal cortex, the OFC, and the caudate and putamen.

Dopaminergic dysfunction in mesocorticolimbic pathways may also contribute to the loss of motivation observed in PD. For example, Parkinson patients are highly susceptible to placebo response, putatively due to endogenous activation of dopaminergic reward systems [32,33]. Dodd et al. [42] reported pathological gambling behavior among a group of Parkinson patients taking dopamine D3 receptor agonists. Voon et al. [158] noted that behaviors such as pathological gambling, hypersexuality, compulsive shopping, and compulsive eating may affect up to 14 percent of Parkinson patients receiving dopamine agonist therapy. The highest concentrations of D2/3 receptors in the human brain are found in mesolimbic regions [145]. Persico et al. [111] administered methylphenidate, a psychostimulant associated with mesocorticolimbic dopamine release, to Parkinson patients and found that they experienced attenuation of the drug-induced "good" feelings experienced by controls. Lemke et al. [71] showed that the DA2/3 receptor agonist pramipexole ameliorates depressive symptomatology including anhedonia in Parkinson patients. These results suggest that dopamine agonists may make an important contribution to the treatment of dPD without the side-effects and drug interactions attributable to anti-depressant pharmacotherapies [70]. Recent research in the psychiatric literature supports the role of mesocorticolimbic dopamine in the pathogenesis and treatment of depression [52].

### 2.9. *Serotonin and dopamine interactions*

In the past few decades, mounting evidence has shown that serotonin and dopamine systems interact in the brain in ways that have implications for the understanding and treatment of depression in PD. In particular, research has shown that serotonin acts via several receptor subtypes to modulate dopaminergic activity in nigrostriatal, mesolimbic, and mesocortical pathways [1,37]. These serotonin receptor subtypes function either to facilitate or inhibit dopaminergic release [2,38]. Midbrain dopaminergic nuclei also exert reciprocal control over the activity of serotonergic neurons [35]. There is evidence that the ability of SSRIs and tricyclic antidepressants to potentiate dopaminergic transmission in the mesocorticolimbic system may contribute to the therapeutic effect of these medications [37]. Larisch et al. [64] found an increase in D2 receptor binding during serotonin re-uptake inhibition in the striatum and anterior cingulate of psychiatric patients with major depression who responded to treatment, suggesting that response to SSRIs may occur in part via the effects of these drugs on dopamine systems. The precise mechanism of action of various antidepressants is not fully understood and research suggests that there is some variability in the effects of different drugs on serotonin receptor subtypes and dopamine transmission [45]. This variability may underlie inconsistencies in the literature regarding anti-depressant efficacy in PD.

## 3. Anxiety in Parkinson's disease

Evidence suggests that up to 40 percent of Parkinson patients have anxiety disturbances including panic disorder, social phobia, generalized anxiety disorder, and anxiety symptoms that do not meet criteria for a specific anxiety disorder [40,98,115,144]. The rate of anxiety in PD patients exceeds that of both the general population and individuals with chronic medical conditions, suggesting that it is not solely a reaction to Parkinson disability.

Earlier studies characterized anxiety in PD as atypical, but more recent research suggests that these cases may fall into the DSM IV Text Revision category of anxiety disorders "not otherwise specified" [115]. In the last several years, investigators have found that non-motor symptoms such as anxiety fluctuate in PD similarly to motor symptoms and often prove as debilitating as motor fluctuations [166,167]. Anxiety tends to occur during the motor "off" state although it can fluctuate independently. Some studies find a correlation between anxiety symptoms and stage of PD [40]. It is uncertain whether in these cases anxiety is a reaction to worsening motor symptoms or whether the two classes of symptoms share a common dopaminergic substrate. Transient symptomatic anxiety or anxiety "not otherwise specified" and syndromal anxiety disorders may constitute separate entities in PD with differing etiologies. Like depression, research has shown that syndromal anxiety disorders may pre-date PD and are more frequent in relatives of PD patients, suggesting that an underlying neurotransmitter aberration may contribute to this co-morbidity [4].

Anxiety in PD often occurs with depression, although the reported frequency of this co- occurrence varies depending upon sample characteristics, diagnostic criteria, and method of assessment. Schiffer et al. [128] applied Research Diagnostic Criteria to depressed patients with PD and depressed patients with multiple sclerosis (MS) and found a higher rate of anxiety and panic disorders in depressed PD patients than in depressed MS patients. Estimates from more recent studies show that between 14 and 65 percent of PD patients have co-morbid diagnoses of anxiety and depression [40,93,102,115]. Starkstein et al. [141] found a significant association between anxiety and sad mood in PD patients that was not influenced by stage of illness.

The rating scales used most frequently to assess anxiety in PD are the Beck Anxiety Inventory, the Hospital Anxiety and Depression Scale, the Zung Self-rating Anxiety Scale and Anxiety Status Inventory, the Spielberger State Trait Anxiety Inventory, and the Hamilton Anxiety Rating Scale. In a recent examination of these scales commissioned by the Movement Disorder Society, Leentjens et al [66] were unable to recommend a single instrument and suggested more research on the topic of anxiety assessment in PD. Their recommendation was based on lack of validation studies in PD, changing criteria that overlap with Parkinson motor and somatic symptoms, and insufficient information on the discriminative properties of these scales.

Abnormalities in monoamine neurotransmitter systems have been implicated in the pathogenesis of anxiety. For example, studies have shown abnormal plasma levels of homovanillic acid, a metabolite of dopamine, in individuals with panic disorder [126]. This condition afflicts between 10 and 30 percent of PD patients [40, 102,144]. Remy et al. [122] used [11C] RTI-32 PET, an in vivo marker of both dopamine and norepinephrine transporter binding, to examine depression and anxiety in PD. Their results suggest that these symptoms are associated with loss of dopaminergic and noradrenergic innervation in the limbic system. Functional polymorphisms in the serotonin transporter gene have also been linked with anxiety in PD [92].

## 4. Parkinsonian apathy

In addition to depression and anxiety, Parkinson patients also experience apathy. Apathy is common in PD, is associated with specific cognitive impairments, and may have a mechanism different from that of depression. In an early study, Starkstein et al. [140] found that 12% of Parkinson patients showed apathy as their primary psychiatric problem while 30% were both apathetic and depressed. Patients with apathy showed significantly more deficits in verbal memory and time-dependent tasks. Levy et al. [73] examined neuropsychiatric and cognitive function in patients with PD and other neurodegenerative diseases. They found that five percent of PD patients had apathy only, 28 percent had depression only, 28 percent had both, and 11 percent had neither apathy nor depression. Apathy but not depression was associated with cognitive impairment. Oguru et al. [105] examined the co-occurrence of apathy and depression in 150 PD patients and found that 60 percent had apathy, 56 percent had depression and 43 percent suffered from both disorders. Apathy was associated with cognitive deficits while depression negatively correlated with emotional well-being, providing evidence that the two disorders are dissociable.

Several studies have found an association between Parkinsonian apathy and frontal-executive dysfunction [56,113,170]. For example, Zgaljardic et al. [170] found that approximately 44% of non-demented PD patients had significant levels of apathy and apathy was related to self-report of depression. Performance on verbal fluency, working memory, and verbal abstraction significantly predicted increasing levels of apathy. Isella [56] conducted neuropsychological assessment and morphometric analysis of MRI scans in PD patients and found a relationship between apathy and performance on tests of executive function but no correlation between apathy scores and frontotemporal atrophy. In a prospective, longitudinal study of apathy in PD, Pedersen et al. [109] found that about 14 percent of patients were apathetic both initially and four years later while 49 percent who were not apathetic at baseline developed the condition four-years later. Compared with those who were not apathetic at either time-point (37 percent), PD patients who developed apathy at follow-up were more demented at baseline, more frequently demented and depressed at follow-up, and had a more rapid decline in motor function. Patients who were apathetic at both time-points were more depressed at baseline and had greater acceleration of motor decline than those who developed apathy at follow-up. These results suggest that the presence of apathy in PD may signal a more widespread and progressive neuropathological process that encompasses motor and cognitive symptoms alike. Other investigators have found an association between motor subtype of PD and non-motor symptoms such as apathy. For example, Reijnders et al. [119] showed that the non-tremor dominant subtype, characterized by bradykinesia, postural instability, gait disorder, and rigidity, is associated with cognitive deterioration, depression, and apathy. In a clinico-pathological study of PD subtypes, Selikhova et al. [133] found that the non-tremor dominant subgroup had a significantly higher mean pathological grading of cortical Lewy bodies than did subgroups with earlier disease onset, predominant tremor, and rapid disease progression without dementia. Furthermore, the non-tremor dominant subtype had more cortical amyloid-beta plaque load and cerebral amyloid angiopathy than did subgroups with earlier disease onset and predominant tremor [133].

Discrepancies in the reported prevalence of Parkinsonian apathy and its co-morbidity with depression are due in part to lack of consensus on a definition of apathy and resultant variability in methods of assessment. While depression is recognized as a disorder by the DSM IV, apathy is not and therefore lacks diagnostic criteria. Furthermore, one of the core features of MDD is anhedonia or loss of interest in daily activities. Apathy scales vary in the extent to which they include anhedonia as a symptom of apathy. Finally, clinical definitions of apathy vary in the extent to which they include cognitive, emotional, and behavioral features in the diagnosis [139].

To address such issues, Starkstein and Leentjens [139] reviewed conceptualizations of apathy as applied to clinical neuropsychiatric practice and called for consensus on diagnostic criteria as well as further validation of assessment scales. Concurrently, the Movement Disorder Society commissioned a study of apathy and anhedonia rating scales in PD. Leentjens et al. [67] reviewed four apathy scales plus the Unified Parkinson's Disease Rating Scale (UPDRS) apathy item and were only able to recommend the Apathy Scale developed by Starkstein et al [140]. This scale targets Parkinson patients, has acceptable psychometric properties, sensitivity to change, a patient-based and caregiver version, and was validated in other patient groups (e.g. stroke and Alzheimer's disease).

In an attempt to develop and standardize diagnostic criteria for apathy in PD, Starkstein et al. [142] administered a structured psychiatric interview and items from the Apathy Scale to PD patients and generated a set of seven diagnostic criteria characterized by high sensitivity and specificity. These criteria include poor to no motivation, diminished goal-directed cognition (e.g. lack of interest in new things), and diminished emotional concomitants of goal-directed behavior (e.g. flat affect, lack of emotional response to positive or negative events). Using this approach, they reported that 32 percent of PD patients in their sample of 164 were apathetic and that apathy was significantly associated with severity of depression and dementia. Apathy without depression or dementia occurred in 13 percent of patients.

Taken together, these studies suggest that the presence of apathy in PD is an ominous clinical sign that may be associated with more rapid progression of motor and cognitive symptoms and possibly accompanied by neuropathological features such as Lewy bodies, beta amyloid plaques, and cerebral amyloid angiopathy. The future of apathy research in PD is dependent upon consensus in diagnostic criteria, the continued development of psychometrically valid and reliable scales, and the use of advanced neuroimaging and autopsy analyses to examine neural correlates of this syndrome.

## 5. Summary and conclusions

Parkinson's disease is accompanied by relatively high rates of depression, and a sizable proportion of patients with PD have anxiety and/or apathy. Because patients are often both anxious and depressed, some investigators have concluded that Parkinson's disease-related depression is atypical, although recent research in psychiatric patients suggests that this co-morbidity may be more common than previously recognized. Considerable evidence suggests that depression in PD is associated with frontal executive dysfunction, including deficits in working memory and set shifting. Some research shows that major depression in PD may be associated with a more widespread pattern of neuropsychological impairment, in contrast to dysthymia which represents an exacerbation of deficits seen in non-depressed Parkinson patients. Depressive disorders may be bellwethers of Parkinson's disease, and there is some evidence that they are part of a complex that is of familial origin. Substantial data indicate that Parkinsonian depression is endogenous rather than reactive; i.e., related to aberrant dopamine, serotonin, and norepinephrine neurotransmission in frontosubcortical pathways. In particular, research commonly identifies abnormalities in the brainstem raphe, locus ceruleus, caudate, thalamus, limbic system, anterior cingulate, OFC, and medial frontal cortex in patients with Parkinsonian depression. Recent studies suggest that mood dysregulation in PD may be linked to aberrant serotonin-dopamine interaction and malfunctioning dopaminergic reward systems in the brain. These observations have led to the development of treatment options for depression that target D2 and D3 receptors. The specific action of various types of antidepressants and the interplay of serotonin and dopamine in the genesis of Parkinsonian depression are important areas of future research.

Emotional disorders associated with idiopathic PD are increasingly recognized as equally or more disabling than the classic motor symptoms. Furthermore, while the treatment paradigms for motor symptomatology are well-established, current understanding of how to treat emotional processing deficits in PD is less advanced. Depression in PD is associated with greater medical and psychiatric co-morbidity and greater healthcare utilization. Specifically, depressed Parkinson patients have significantly higher rates of dementia, psychosis, and stroke than non-depressed Parkinson patients and depressed patients without PD [24]. Chen and colleagues [24] also reported that depressed Parkinson patients are significantly more likely than non-depressed Parkinson patients to have medical and psychiatric hospitalizations, in addition to having more out-patient visits.

The affective symptomatology of idiopathic PD is costly from economic, social, and personal standpoints. Neuropsychiatric assessment and monitoring of PD patients is essential in providing optimal care of these patients. In particular, the evaluation of patients with PD should include the administration of neuropsychiatric rating scales to assess the three most common conditions affecting PD patients: depression, anxiety, and apathy. We have discussed such instruments above and for screening purposes in a clinic setting suggest self-report measures that have been used and preferably validated in PD. Specific recommendations based on the literature reviewed include the 15 item Geriatric Depression Scale [134] or the Beck Depression Inventory (version IA [8] or version II [which more closely reflects DSM criteria but has not been as widely used in PD [9], the Beck Anxiety Inventory [7] or the State Trait Anxiety Inventory [137] (but see Leentjens [66] for critique), and the Apathy Scale [140]. These self-report measures are easily completed in the clinic and provide screening information to be used in treatment planning. Consultation with and referral to mental health professionals should be considered in PD patients suspected of having affective disorders. Treatment of emotional dysfunction in PD is an area of on-going research and thus it is essential for physicians involved in the care of these patients to keep abreast of clinical trial outcomes. While currently available anti-depressants and anxiolytics may help to alleviate mood disturbance in PD patients, there are no tested or validated treatments targeting apathy in PD. Nevertheless, clinicians should assess and monitor this condition as it may signal a more aggressive disease process involving both motor and non-motor function. Lastly, continued basic and translational research into the etiology and treatment of emotional disorders in patients with Parkinson's disease, and their relationships to cognitive function and quality of life, will benefit current and future sufferers of this devastating degenerative neuropsychiatric disease.

## Acknowledgements

Supported by NIMH/NINDS grant R01MH78228 and the Department of Veterans Affairs. We thank Michelle Lineberry and Sherry Williams for their editorial assistance.
